# Characterization of Two Variants at Met 1 of the Human *LDLR* Gene Encoding the Same Amino Acid but Causing Different Functional Phenotypes

**DOI:** 10.3390/biomedicines9091219

**Published:** 2021-09-14

**Authors:** Rafael Graça, Rafael Fernandes, Ana Catarina Alves, Juliane Menezes, Luísa Romão, Mafalda Bourbon

**Affiliations:** 1 Departamento de Promoção da Saúde e Prevenção de Doenças Não Transmissíveis, Instituto Nacional de Saúde Doutor Ricardo Jorge, 1600-609 Lisbon, Portugal; rafael.graca@insa.min-saude.pt (R.G.); catarina.alves@insa.min-saude.pt (A.C.A.); 2 BioISI—Biosystems & Integrative Sciences Institute, Faculdade de Ciências, Universidade de Lisboa, 1749-016 Lisbon, Portugal; rafael.fernandes@insa.min-saude.pt (R.F.); juliane.menezes@insa.min-saude.pt (J.M.); luisa.romao@insa.min-saude.pt (L.R.); 3 Departamento de Genética Humana, Instituto Nacional de Saúde Doutor Ricardo Jorge, 1600-609 Lisbon, Portugal

**Keywords:** initiation codon, familial hypercholesterolemia, *LDLR*, functional characterization, ACMG classification

## Abstract

Familial hypercholesterolemia (FH) is the most common genetic disorder of lipid metabolism, characterized by increased levels of total and LDL plasma cholesterol, which leads to premature atherosclerosis and coronary heart disease. FH phenotype has considerable genetic heterogeneity and phenotypic variability, depending on LDL receptor activity and lifestyle. To improve diagnosis and patient management, here, we characterized two single nucleotide missense substitutions at Methionine 1 of the human *LDLR* gene (c.1A>T/p.(Met1Leu) and c.1A>C/p.(Met1Leu)). We used a combination of Western blot, flow cytometry, and luciferase assays to determine the effects of both variants on the expression, activity, and synthesis of *LDLR*. Our data show that both variants can mediate translation initiation, although the expression of variant c.1A>T is very low. Both variants are in the translation initiation codon and codify for the same amino acid p.(Met1Leu), yet they lead to different levels of impairment on *LDLR* expression and activity, corroborating different efficiencies of the translation initiation at these non-canonical initiation codons. The functional data of these variants allowed for an improved American College of Medical Genetics (ACMG) classification for both variants, which can allow a more personalized choice of the lipid-lowering treatment and dyslipidemia management, ultimately improving patients’ prognosis.

## 1. Introduction

Familial hypercholesterolemia (FH) (OMIM 143890) is an autosomal disorder of lipid metabolism characterized by increased plasma cholesterol levels and lipid accumulation in arteries and tendons, promoting premature atherosclerosis and coronary heart disease (CHD) [1]. FH is one of the most common genetic disorders with a frequency of around 1:250 in most populations, according to a recent meta-analysis [2]. The FH phenotype can be caused by pathogenic variants in the three FH-associated genes (*APOB*, *LDLR,* and *PCSK9*) or by pathogenic variants in several phenocopies or alternative molecular etiologies (*ABCG5/8*, *APOE*, *LDLRAP1,* and *LIPA*), or it can even be due to a polygenic cause [3,4,5,6]. Although the underlying genetics of the FH phenotype is complex, more than 90% of FH cases result from *LDLR* defects associated with an autosomal dominant inheritance pattern [3,4]. Up to date, there are more than 2300 *LDLR* variants exclusively identified in FH patients [7]. Another key feature of FH genetics is the phenotypic variability [3,8]; there is a diversity of phenotypes between groups sorted by causative gene and mutation type, and it is not uncommon to observe a wide range of lipid levels among carriers of an identical FH-causing mutation [3,8]. Moreover, the mutation type and the percentage of activity of *LDLR* variants can modulate the patients’ phenotype and response to lipid therapy [4,8]. *LDLR* variants can be primarily divided into null variants and defective variants, with complete or partial loss of function, respectively. Several studies showed an association between higher levels of LDL cholesterol and null variants compared with defective variants [9,10,11]. Additionally, the type of mutation can modulate response to statin therapy, as shown in Brazilian and Spanish cohorts, where null variants were associated with the lowest decrease of LDL levels upon treatment [12,13]. *LDLR* mutations can be functionally classified into five groups: class I—no protein synthesis (null variants); class II—partial or complete retention of the protein at the endoplasmic reticulum; class III—defective binding; class IV—defective internalization or endocytosis; class V—defective recycling [14]. Although the functional characterization is important for a correct patient diagnosis and management, less than 10% of all variants described in the *LDLR* gene have been functionally characterized. To improve diagnosis and patient management, here, we report two different missense variants (c.1A>T, p.(Met1Leu) and c.1A>C, p.(Met1Leu)) in the *LDLR* initiation codon, exhibiting distinct functional phenotypes.

## 2. Materials and Methods

### 2.1. Clinical Study

The Portuguese FH Study is a research project continuously running since 1999 with the aim of identifying the genetic cause of hypercholesterolemia in individuals with a clinical diagnosis of FH [15,16,17]. The study protocol and database have been approved by the National Institute of Health Ethics Committee and the National Data Protection Commission, respectively. Written informed consent was obtained from all participants before their inclusion in the study. Fasting blood samples were collected from all individuals at the time of their inclusion in the study. The biochemical characterization of lipids and lipoproteins was performed in a Cobas Integra 400 plus (Roche, Risch-Rotkreuz, Switzerland) by enzymatic colorimetric and immunoturbidimetric methods. All healthy individuals from whom the LDL used in the experiments was extracted followed the same exact procedure as any participant of the Portuguese FH Study.

### 2.2. Plasmid Constructs

For Western blot and cytometry assays, the two missense variants under study, NM_000527.5:c.1A>T and NM_000527.5:c.1A>C, were individually introduced into the human *LDLR* cDNA (NM_000527.5) in the mammalian expression vector pcDNA3 under control of an SV40 promoter and enhancer. For luminometry assays, the promoter and the 5′ untranslated region (5′UTR) of *LDLR* (−319 bp upstream from ATG) were cloned upstream the Firefly Luciferase (FLuc) coding region in the pGL4.10 reporter plasmid (Promega, Madison, WI, USA), obtaining the *LDLR*_pGL4-WT construct. Both plasmids were subjected to oligonucleotide site-directed mutagenesis using the NZYMutagenesis kit (NZYTech, Lisbon, Portugal), according to the manufacturer’s instructions. For every variant, the presence of the correct variant and the integrity of the region was confirmed by direct Sanger sequencing.

### 2.3. Cell Culture and Transfection

*LDLR*-deficient CHO-ldlA7 cells (Chinese hamster ovary cell line ldlA7), kindly provided by Dr. Monty Krieger, Massachusetts Institute of Technology, Cambridge, MA, were cultured in F-12 Nut Mix medium supplemented with 10% (*v*/*v*) fetal bovine serum (FBS, Invitrogen, Waltham, MA, USA), 100 units/mL penicillin, and 100 μg/mL streptomycin, and cells were grown at 37 °C in a humidified incubator containing 5% CO_2_.

For Western blot and cytometry assays, cells were seeded in 6- or 24-well culture plates at 80% confluence (48 h) and were transfected with plasmids containing wild-type or mutant *LDLR* variants using Lipofectamine 2000 Transfection reagent (Invitrogen, Waltham, MA, USA) following manufacturer’s instructions. *LDLR* expression and LDL binding and internalization were assessed 24 h after transfection.

For luminometry assays, transient transfections were performed using Lipofectamine 2000 Transfection Reagent (Invitrogen, Waltham, MA, USA) following the manufacturer’s instructions in 35-mm plates. Cells were co-transfected with 750 ng of the test DNA construct corresponding to the *LDLR*_pGL4-WT or its derivative plasmids and 500 ng of the pRL-TK plasmid (Promega, Madison, WI, USA), which encodes *Renilla* luciferase (RLuc) as an internal control, and then harvested after 24 h.

### 2.4. Western Blot Analysis

Protein expression was determined for whole-cell extracts and fractionated by electrophoresis using NuPAGE™ 4%–12% Bis-Tris gel (Invitrogen, Waltham, MA, USA) and NuPAGE™ MOPS SDS Running Buffer (Invitrogen, Waltham, MA, USA) for semi-quantitative immunoblotting. PVDF membranes were immunostained overnight at 4 °C with a specific rabbit polyclonal antibody to the human LDL receptor (Progen Biotechnik, Heidelberg, Germany) and a monoclonal anti-α-tubulin antibody (Boster Biological Technology, Pleasanton, CA, USA) for protein loading control. After incubation with appropriate secondary antibodies, proteins were detected by chemiluminescence using Pierce™ ECL Plus Western Blotting Substrate (Thermo Scientific, Waltham, MA, USA) on a ChemiDoc (BioRad, Hercules, CA, USA). Relative band intensities of mature (≈160 kDa) and precursor (≈120 kDa) forms of *LDLR* protein were quantified by densitometric analysis using Image Lab Software (BioRad, Hercules, CA, USA) and normalized to levels of α-tubulin.

### 2.5. LDL Isolation and Labeling

Plasma used for lipoprotein purification was collected from healthy individuals’ blood after 30 min centrifugation at 2465× *g* at room temperature (5810R, Eppendorf, Hamburg, Germany). For all samples, plasma density was adjusted to 1.21 g/mL with potassium bromide (KBr), and PBS buffer was added to obtain a clear separation between lipid fractions. LDL was isolated through ultracentrifugation carried out in a TST 41–14 rotor at 225,000× *g* for 19 h at 4 °C (Centrikon T-21X0, Kronton, Munich, Germany). The intermediate orange band corresponding to LDL was collected and stored at 4 °C.

LDL was fluorescently labeled with fluorescein isothiocyanate (FITC). LDL was loaded in a 0.1 M NaHCO3 (pH 9.0) pre-equilibrated Sephadex G-25 column and then mixed under constant agitation with 10 μL FITC (2 mg/mL in DMSO) per lipoprotein milliliter at room temperature for 2 h. After incubation, free FITC was removed by gel filtration on a Sephadex G-25 column equilibrated with PBS EDTA-free buffer. Lipoprotein quantification was determined by the Pierce BCA protein assay.

### 2.6. LDLR Expression and Activity (Lipoprotein Binding and Internalization) by FACS

*LDLR* cell surface expression was measured by FACS using mouse anti-human-*LDLR* (1:100; Progen Biotechnik, Heidelberg, Germany) as primary antibody and Alexa Fluor 488-conjugated goat anti-mouse IgG (1:200; Molecular Probes, Eugene, OR, USA) as the secondary antibody. Briefly, cells were incubated at 4 °C, overnight with the primary antibody after fixing (10 min in 4% paraformaldehyde) and blocking (1 h with PBS-5% FBS) steps. Next, cells were incubated for 1 h at room temperature with the secondary antibody. For each sample, the fluorescence of 15,000 events was acquired for data analysis, and measurements were performed in triplicate. All washes were done with PBS-1% BSA.

For quantification of *LDLR* activity, cells were seeded in 24-well culture plates and transfected, as previously described. Briefly, 24 h after transfection, cells were incubated for 3 h at 37 °C or 4 °C with 20 μg/mL FITC-LDL to determine LDL internalization or LDL-*LDLR* binding, respectively. After incubation, cells were washed three times, fixed for 10 min in 4% paraformaldehyde, and washed again three times. The amount of internalized LDL was determined by adding Trypan blue solution to a 0.2% final concentration (Sigma-Aldrich, Steinheim, Germany) to the samples. This dye quenches external fluorescence, eliminating the signal of non-internalized *LDLR*-LDL complexes, allowing the determination of the intensity of the remaining fluorescent particles inside the cells. Fluorescence intensities were measured by flow cytometry in a Facscalibur Flow cytometer. For each sample, the fluorescence of 15,000 events was acquired for data analysis, and measurements were performed in triplicate. All washes were done with PBS-1% BSA.

### 2.7. Luminometry Assay 

Cell lysis was performed with Passive Lysis Buffer (Promega). The cell lysates were used to determine relative FLuc and RLuc activities using the Dual-Luciferase^®^ Reporter Assay System (Promega), according to the manufacturer’s instructions, on a GloMax^®^ 96 Microplate Luminometer (Promega). The collected data was expressed in arbitrary light units. Luciferase activity was obtained by normalizing FLuc to RLuc luminescence for each sample, and each value was derived from three independent experiments.

### 2.8. American College of Medical Genetics (ACMG) Classification and In Silico Analysis

Both variants were classified according to the Clinical Genome resource FH variant curation expert panel specifications for the ACMG/AMP Variant Interpretation Guidelines [18]. Additionally, the potential effects of the two variants were evaluated by 5 software tools: PROVEAN [19], SIFT [20], PolyPhen2 [21], MutationTaster [22], and REVEL [23].

### 2.9. Statistical Analysis

Results are expressed as mean ± standard deviation. The Student’s *t*-test was used for the estimation of statistical significance. Significance for statistical analysis was defined as a *p* < 0.001.

## 3. Results

### 3.1. In Silico and ACMG Classification

The bioinformatics prediction for the pathogenicity of the two variants displays conflicting classification between the different software but not among themselves (Table 1).

Initially, variants c.1A>T, p.(Met1Leu), and c.1A>C, p.(Met1Leu) were classified as VUS (variant of uncertain significance) and Likely Pathogenic, respectively. Once the functional data were integrated into the algorithm, the classification has improved to Likely Pathogenic and Pathogenic (Table 2).

### 3.2. Functional Profiling of Variants c.1A>T and c.1A>C

**Figure 1:**
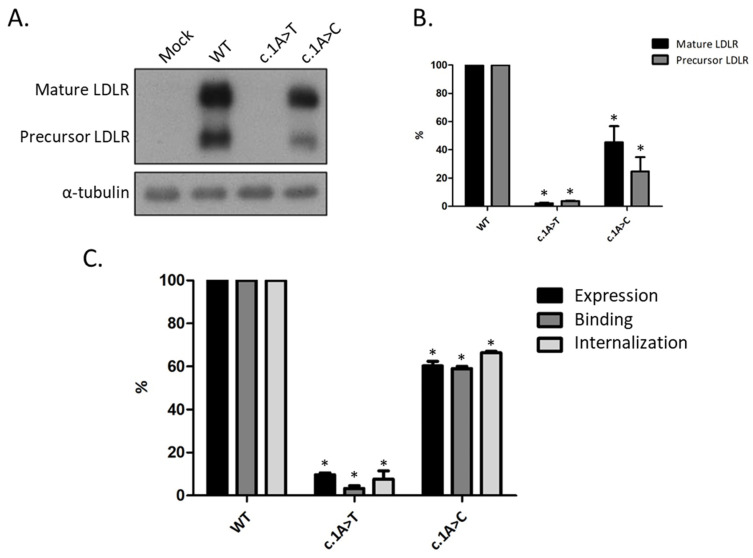
Expression and activity of *LDLR* in CHO-ldlA7 cells transfected with wild-type (WT), c.1A>T, p.(Met1Leu), or c.1A>C, p.(Met1Leu) variants. After 24 h of overexpression, cells were lysed, and protein extracts were analyzed (**A**) by Western blot, as previously described in Methods. (**B**) The relative band intensities of both mature (black bars) and precursor (grey bars) *LDLR* forms were calculated, respectively, as the ratio of 160 kDa or 120 kDa *LDLR* band intensity to that of α-tubulin. (**C**) Black bars—*LDLR* expression; Dark grey bars—LDL(FITC)-*LDLR* binding after 3 h incubation at 4 °C; Light grey bars—LDL(FITC) internalization after 3 h incubation at 37 °C. Geometric fluorescence intensity of 15,000 events was acquired in a Facscalibur Flow cytometer, as mentioned above. All values represent the mean of triplicate independent determinations (*n* = 3); error bars represent ± SD. * *p* < 0.001 compared to *LDLR* WT using a two-tailed Student’s *t*-test.

Aiming to understand the functional consequences of both variants, protein levels from each variant were analyzed by Western blot (Figure 1A), and relative levels of *LDLR* expression were determined by quantitative densitometry, using α-tubulin protein expression as an internal control (Figure 1B). This analysis revealed different mature and precursor *LDLR* expression levels between the two variants (Figure 1A). *LDLR* variant c.1A>T produces almost no protein, showing a similar protein profile to the mock control. Whereas in *LDLR* variant c.1A>C, both mature and precursor forms of *LDLR* were detected, being the expression lower compared to the wild-type (WT) receptor (Figure 1B).

To quantify the expression and activity of both *LDLR* variants, CHO-ldlA7 cells were transiently transfected with WT *LDLR*, c.1A>T, p.(Met1Leu), or c.1A>C, p.(Met1Leu) constructs to determine the ability of the receptor to bind and internalize labeled LDL. Results for cell surface *LDLR* expression, LDL (FITC)-*LDLR* binding, and internalization are illustrated in Figure 1C. WT transfection was used for normalization of results, and it represents 100% of expression and activity. Both variants exhibit expression and activity levels below 70% of the WT *LDLR*; therefore, they are considered to affect *LDLR* function. Cut-offs for this limit were used from Chora et al. (2021) [18]. The two variants exhibit cell surface expression lower than WT *LDLR*, and consequently, binding and internalization levels are also diminished. Variant c.1A>T shows expression and activity levels below 10%, whereas variant c.1A>C has expression and activity levels around 60%.

**Figure 2:**
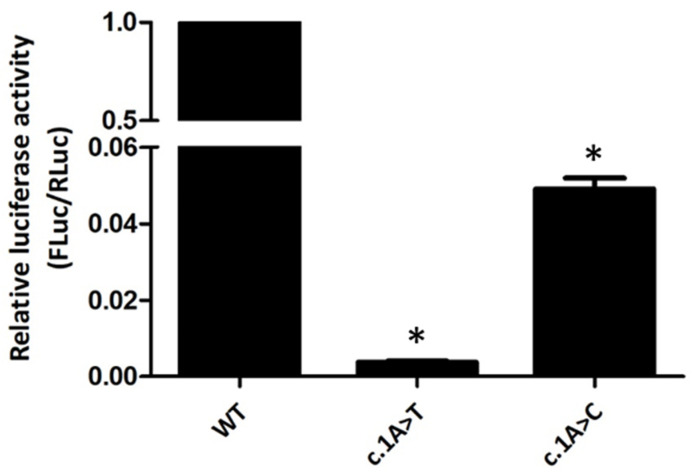
Relative luciferase activity of *LDLR*_pGL4 constructs with c.1A>T, p.(Met1Leu) or c.1A>C, p.(Met1Leu) variants. CHO-ldlA7 cells were transiently co-transfected with each one of the *LDLR*_pGL4 constructs (containing Firefly Luciferase—FLuc) and with the pRL-TK plasmid encoding the *Renilla* Luciferase (RLuc). Cells were lysed 24 h later, and the luciferase activity was measured by luminometry assays. FLuc activity values were normalized to RLuc activity to control for transfection efficiency. Relative luciferase activity of the *LDLR*_pGL4-WT (WT) construct was defined as one. All values represent the mean of triplicate independent determinations (*n* = 3); error bars represent ± SD. * *p* < 0.001 compared to *LDLR* WT using a two-tailed Student’s *t*-test.

Additionally, luminometry assays were performed to check the effect of the two variants in protein synthesis (Figure 2). Relative luciferase activity of constructs pGL4-c.1A>T and pGL4-c.1A>C is 0.5% and 5% of the normal control, respectively. Thus, both c.1A>T and c.1A>C *LDLR* variants repress protein expression of the downstream reporter gene, although with different intensities.

### 3.3. Phenotype of Patients with Variant c.1A>C, p(Met1Leu)

Only the variant c.1A>C is identified in the Portuguese FH Study in four different families exhibiting an autosomal dominant inheritance pattern. A total of eight individuals carries this variant—four index cases and four relatives (Table 3). The eight carriers present untreated values of total and LDL cholesterol (LDL-C) ranging from 288 to 491 mg/dL and from 222 to 392 mg/dL, respectively, in adults. In children, total cholesterol and LDL-C range from 174 to 284 mg/dL and from 108 to 226 mg/dL, respectively.

## 4. Discussion

Both *LDLR* variants (c.1A>T and c.1A>C) are predicted to change Methionine (AUG) to Leucine [UUG or CUG, respectively; p.(Met1Leu)]. Here, we investigated the functional consequences of these two *LDLR* variants. Our data show that both variants are able to mediate translation initiation, although the expression of variant c.1A>T is very low. Translation has been traditionally considered to initiate at the universal AUG start codon. However, due to the advent of the ribosome profiling technique [24], it is now clear that many non-AUG codons can function as non-canonical start codons [25]. Accordingly, here we show that the UUG and CUG Leucine codons are able to function as translation initiation codons for the *LDLR* protein. Our data also show that these two Leucine codons mediate translation initiation with different efficiencies, being the UUG codon a much less efficient initiator. Indeed, these results are in accordance with what has been described, showing that, in eukaryotes, the substitution of the initiating codon CUG for an alternate Leucine-encoding UUG codon drastically reduces protein expression [26]. Moreover, the CUG codon is the most prevalent non-AUG start codon found in upstream open reading frames [27]. Although the key eukaryotic initiation factor (eIF), eIF2, does not significantly bind other tRNAs besides Met-tRNA_i_^Met^ [25], it is known that other eIFs can substitute eIF2 for initiation at non-AUG codons. eIF2A, for instance, can deliver Leu-tRNA^CUG^ and even Met-tRNA_i_^Met^ to initiate translation at non-AUG start codons in a GTP-independent manner [28,29]. Accordingly, depletion of eIF2A does not significantly impair global protein synthesis, neither affects translation initiation at AUGs, but has a negative impact on initiation at CUG or UUG codons [28,29]. These pieces of evidence well support the contrasting in vitro phenotypes observed between the two variants regarding *LDLR* expression. As shown, the construct with the variant c.1A>T has residual *LDLR* expression and consequently very low LDL binding and internalization abilities. Nonetheless, the construct with the variant c.1A>C partially expresses *LDLR*, and the binding and internalization activities are coherently affected. Moreover, our results from the FLuc-based reporter constructs also support these data. However, the expression of these constructs is significantly lower than the one from the constructs with the *LDLR* open reading frame (ORF) (5% versus 60% and 0.5% versus 10% for c.1A>C and c.1A>T, respectively; Figure 1C and Figure 2). One possible explanation for this discrepancy could be the existence of RNA secondary structures downstream the start codons that could favor recognition of the non-canonical, UUG and CUG, codons on the pcDNA3 constructs (*LDLR* ORF) when compared to the pGL4 constructs (Luciferase ORF). This idea is supported by previous reports showing that recognition of non-canonical codons by the scanning ribosomes is favored by GC-rich downstream regions that potentiate the formation of hairpin-like structures. These secondary structures are thought to slow down the ribosomal scanning process, giving time for translation initiation to occur on sub-optimal codons [30,31]. Accordingly, the mRNA secondary structure obtained from mFOLD (http://www.unafold.org; accessed on 7 February 2021) using the first 100 nucleotides of the *LDLR* coding sequence is more stable than the one corresponding to the first 100 nucleotides of the Luciferase ORF.

Considering the functional data gathered on the two variants, variant c.1A>T is classified as an *LDLR* class I mutation, with no protein synthesis (null variant), whereas variant c.1A>C phenotype is similar to *LDLR* class II mutations, with partial retention of the immature protein at the endoplasmic reticulum [14]. These distinct phenotypes, a null and a defective allele type variant, highlight the importance of functionally characterizing and classifying *LDLR* variants. Carriers of a null allele variant present higher LDL values and higher rates of premature CHD than a defective allele carrier [10,32]. As such, functional characterization is important for cardiovascular risk stratification. Considering our in vitro results, a distinctive severity of FH phenotype in carriers of these variants is expected, with carriers of the variant c.1A>C exhibiting a milder phenotype compared to c.1A>T carriers.

According to ACMG/AMP guidelines for the classification of genetic variants, c.1A>T and c.1A>C are classified, respectively, as likely pathogenic and pathogenic variants, taking into consideration the functional study results [18]. However, functionally, they have different performances, as described above. ACMG classification classifies a variant as likely pathogenic or pathogenic or likely benign or benign but does not characterize the level to which the variant affects the protein, which can be determined only with functional studies.

Whereas the variant c.1A>T was first described in 1996 in a compound heterozygous (CH) patient from South Africa [33], variant c.1A>C was first identified in 2001 in a heterozygous patient from Spain (no lipid information provided) [34]. As expected for an FH homozygous or compound heterozygous individual, the CH patient carrying the variant c.1A>T exhibits extremely high levels of total and LDL cholesterol (LDL-C), 719 mg/dL and 669 mg/dL, respectively. Additionally, the variant c.1A>T is also identified in two first degree relatives of the CH patient with ages of 36 and 18 years old, presenting values of total and LDL-C of 363 mg/dL and 305 mg/dL and 305 mg/dL and 232 mg/dL, respectively. Unfortunately, no data on lipid-lowering treatment is provided for any of these individuals. To date, only the variant c.1A>C is identified in the Portuguese FH Study in four different families exhibiting an autosomal dominant inheritance pattern. As reported in the results, the eight carriers present a wide range of untreated values of total and LDL-C, showing the already mentioned heterogeneity in phenotypes presented by carriers of the same variant. There are several cholesterol modulators from age, body mass index, diet, physical exercise, and co-occurrence of other disorders and treatments that can account for this phenotypical heterogeneity. In this case, since the variants under study are in the *LDLR* translation initiation codon and originate a non-canonical initiator, we can speculate that some factors involved in non-canonical translation initiation may also be modulating it with different efficiencies among patients, leading to distinct phenotypes.

It is known that the type of mutation can modulate the response to statin therapy, where null variants have the lowest decrease of LDL levels upon treatment [12,13]. In adults, the lowest observed values of total and LDL-C (227 mg/dL and 149 mg/dL) correspond to an individual undergoing statin therapy, who before treatment had 400 mg/dL of total cholesterol (LDL-C values before treatment not known). The child of this individual also undergoing statin therapy shows a reduction of 23% and 30% of total and LDL-C, i.e., from 335 mg/dL and 250 mg/dL to 257 mg/dL and 174 mg/dL, respectively. This good response to treatment goes to the encounter of our results, to the extent that considering the mechanism of action of statins, theoretically, carriers of putative null variants should not greatly benefit from this therapy. However, from carriers of a variant with partial activity, such as c.1A>C, a good response to statin treatment is expected, as observed in these individuals. Both c.1A>C and c.1A>T variants exert their pathogenicity by means of low expression of *LDLR*; the second one is close to null expression. Although the expression is reduced, the activity of the synthesized receptors is normal, and so they play their role in the clearance of LDL from circulation. This way, the effect of statin therapy, which blocks cholesterol biosynthesis and promotes the hepatic expression of *LDLR* [35], should have a more beneficial impact on carriers of variant c.1A>C compared to c.1A>T carriers. 

## 5. Conclusions

In conclusion, we have characterized 2 different *LDLR* missense variants in the translation initiation codon, which codify for the same amino acid p.(Met1Leu) but have a distinct impact on *LDLR* expression and activity. The two variants exhibit different functional behaviors: cytometry and luciferase assays show that variant c.1A>T/p.(Met1Leu) acts like a null variant with extremely low levels of protein expression and consequent null activity; conversely, variant c.1A>C/p.(Met1Leu) is able to initiate translation with a higher efficiency, which renders a significant *LDLR* activity to this variant. Our data gives new functional insight into these two variants, leading to an improved ACMG classification, which can be used in the choice of the lipid-lowering treatment and dyslipidemia management for a more personalized approach, which, in the end, can lead to a better patient prognosis. With this work, we have also contributed with evidence to the function of non-canonical initiation codons in translation initiation. However, additional experiments are required to fully unveil the underlying mechanism through which mRNA translation initiation is affected.

## Figures and Tables

**Table 1:** In silico results obtained by the different bioinformatics tools.

Variant	PROVEAN	SIFT	Polyphen-2	Mutation Tester 2	REVEL
c.1A>T, p.(Met1Leu)	Neutral	Damaging	Benign	disease causing	0.638
c.1A>C, p.(Met1Leu)	Neutral	Damaging	Benign	disease causing	0.638

**Table 2:** ACMG/AMP classification with and without the functional data.

Variant	ACMG Classification without FS	Points without FS	ACMG Classification after FS *
c.1A>T, p.(Met1Leu)	VUS	PM2, PVS1_Moderate and PP4	Likely pathogenic
c.1A>C, p.(Met1Leu)	Likely pathogenic	PM2, PVS1_Moderate PP1_Moderate, PP4, PS4_Supporting	Pathogenic

FS—Functional study; VUS—variant of uncertain significance; * Classification with additional PS3 point.

**Table 3:** Carriers of variant c.1A>C, p.(Met1Leu) found in the Portuguese FH Study.

Family	Age at Referral	Sex	Total-C * [mg/dL]	LDL-C * [mg/dL]	HDL-C * [mg/dL]	TG * [mg/dL]	BMI (Kg/m^2^)	LLT
1—Index	22	F	439	297	115	135	NK	No
2—Index	38	M	288	222	48	90	NK	No
2	9	F	174	108	52	53	NK	No
3—Index	2	M	281	226	47	93	16.8	No
3	3	M	284	220	51	95	NK	No
3	32	F	491	392	77	110	NK	No
4—Index	12	M	257	174	68	32	22.6	Atorvastatin
4	41	F	227	149	63	50	NK	Atorvastatin

* Values at referral for a genetic test. TG—triglycerides; BMI—body mass index; LLT—lipid-lowering therapy; NK—not known.

## Data Availability

The data that support the findings of this study are available from the corresponding author upon reasonable request.
